# Consensus statement for treatment protocols in pressurized intraperitoneal aerosol chemotherapy (PIPAC)

**DOI:** 10.1515/pp-2022-0102

**Published:** 2022-03-01

**Authors:** Olivia Sgarbura, Clarisse Eveno, Mohammad Alyami, Naoual Bakrin, Delia Cortes Guiral, Wim Ceelen, Xavier Delgadillo, Thanh Dellinger, Andrea Di Giorgio, Amaniel Kefleyesus, Vladimir Khomiakov, Michael Bau Mortensen, Jamie Murphy, Marc Pocard, Marc Reymond, Manuela Robella, Koen P. Rovers, Jimmy So, S.P. Somashekhar, Clemens Tempfer, Kurt Van der Speeten, Laurent Villeneuve, Wei Peng Yong, Martin Hübner

**Affiliations:** Department of Surgical Oncology, Cancer Institute of Montpellier, University of Montpellier, Montpellier, France; IRCM, Institut de Recherche en Cancérologie de Montpellier, INSERM U1194, Université de Montpellier, Institut régional du Cancer de Montpellier, Montpellier, France; Department of Digestive and Oncological Surgery, University of Lille, Claude Huriez University Hospital, Lille, France; Department of General Surgery and Surgical Oncology, Oncology Center, King Khalid Hospital, Najran, Saudi Arabia; Department of General Surgery & Surgical Oncology, Centre Hospitalier Lyon-Sud, Hospices Civils de Lyon, Pierre-Bénite, France; Lyon University 1, EA 3738 CICLY, Lyon, France; Department of GI Surgery, Ghent University Hospital, Ghent, Belgium; Centre Médico Chirurgical Volta, Unité Spécialisée de Chirurgie, La Chaux-de-Fonds, Switzerland; Department of Gynecologic Oncology, City of Hope National Medical Center, Duarte, CA, USA; Peritoneal and Retroperitoneal Surgical Unit, Fondazione Policlinico Universitario Agostino Gemelli IRCCS, Rome, Italy; Department of Visceral Surgery, Lausanne University Hospital CHUV, University of Lausanne (UNIL), Lausanne, Switzerland; P.A. Hertsen Moscow Research Oncological Institute – Branch of the National Medical Research Center of Radiology, Moscow, Russia; Department of Surgery, Odense Pancreas Center (OPAC) & Odense PIPAC Center (OPC), Odense University Hospital, Odense, Denmark; Academic Surgical Unit, Imperial College Healthcare NHS Trust, London, UK; Université de Paris, INSERM, U1275 CAP Paris-Tech, Paris, France; Hepato-Biliary-Pancreatic Gastrointestinal Surgery and Liver Transplantation, Pitié Salpêtrière Hospital, AP-HP, Paris, France; Department of Surgery, University of Tübingen, Tübingen, Germany; Unit of Surgical Oncology, Candiolo Cancer Institute-FPO, IRCCS, Turin, Italy; Department of Surgery, Catharina Cancer Institute, Eindhoven, The Netherlands; Division of Surgical Oncology, National University Cancer Institute, Singapore, Singapore; Department of Surgical Oncology, Manipal Comprehensive Cancer Center, Manipal Hospital, Bangalore, India; Department of Obstetrics and Gynecology and Therapy Center for Peritoneal Carcinomatosis, Marien Hospital Herne, Ruhr-Universität Bochum, Herne, Germany; Department of Surgical Oncology, Ziekenhuis Oost-Limburg, Genk, Belgium; Department of Public Health, Clinical Research and Epidemiology, Hospices Civils de Lyon, Lyon, France; Cancer Science Institute of Singapore, National University of Singapore, Singapore, Singapore

**Keywords:** cisplatin, doxorubicin, oxaliplatin, peritoneal metastasis, PIPAC, standardisation, treatment protocol

## Abstract

**Objectives:**

Safe implementation and thorough evaluation of new treatments require prospective data monitoring and standardization of treatments. Pressurized intraperitoneal aerosol chemotherapy (PIPAC) is a promising alternative for the treatment of patients with peritoneal disease with an increasing number of suggested drug regimens. The aim was to reach expert consensus on current PIPAC treatment protocols and to define the most important research topics.

**Methods:**

The expert panel included the most active PIPAC centers, organizers of PIPAC courses and principal investigators of prospective studies on PIPAC. A comprehensive literature review served as base for a two-day hybrid consensus meeting which was accompanied by a modified three-round Delphi process. Consensus bar was set at 70% for combined (strong and weak) positive or negative votes according to GRADE. Research questions were prioritized from 0 to 10 (highest importance).

**Results:**

Twenty-two out of 26 invited experts completed the entire consensus process. Consensus was reached for 10/10 final questions. The combination of doxorubicin (2.1 mg/m^2^) and cisplatin (10.5 mg/m^2^) was endorsed by 20/22 experts (90.9%). 16/22 (72.7%) supported oxaliplatin at 120 with potential reduction to 90 mg/m^2^ (frail patients), and 77.2% suggested PIPAC-Ox in combination with 5-FU. Mitomycin-C and Nab-paclitaxel were favoured as alternative regimens. The most important research questions concerned PIPAC conditions (n=3), standard (n=4) and alternative regimens (n=5) and efficacy of PIPAC treatment (n=2); 8/14 were given a priority of ≥8/10.

**Conclusions:**

The current consensus should help to limit heterogeneity of treatment protocols but underlines the utmost importance of further research.

## Background

Pressurized intraperitoneal aerosol chemotherapy (PIPAC) has been proposed in 2011 as novel modality for the palliative treatment of peritoneal metastases (PM) of various origins. Two empirical protocols have been suggested by the pioneer group, namely (I) oxaliplatin (Ox) at 92 mg/m^2^ body surface i.e. 20% of the Elias regimen for hyperthermic intraperitoneal chemotherapy (HIPEC) for PM of colorectal and appendicular origin and (II) the combination treatment of doxorubicin (D) 1.5 mg/m^2^ and cisplatin (C) 7.5 mg/m^2^ for all other entities representing each ≤10% of current HIPEC doses [1], [2], [3] (Table 1). Feasibility, safety and excellent tolerance has been demonstrated repeatedly along with promising clinical outcomes, essentially by the use of these two drug regimens [4], [5], [6].

**Table 1:** Overview on drug regimens used for PIPAC, HIPEC, and systemic treatment.

	PIPAC dose, mg/m^2^	IV dose, mg/m^2^	HIPEC dose, mg/m^2^	NIPS dose, mg/m^2^	PIPAC/IV %	PIPAC/HIPEC, %
Ox	46–135	85	200–460	85–130	50–160	10–30
Dox	1.5–2.1	15	15^a^	NA	10–13	10
Cis	7.5–10.5	75	50^a^	NA	10–13	15–21
MMC	1.5 (14 mg total dose)^c^	20	10–35	NA	43–75	4–15
Iri	20^c^	125–180	200	NA	11–16	10 (0.1)
Ptx	30^b^	135–175	60–175	20–80	17–22	17–50
Nab-Ptx	112,5	125	NA	NA	90	NA

^a^Doses only used for the combination of drugs, not for the independent use of each drug; ^b^dose based only on animal studies; ^c^doses based on expert opinion and not published in the literature.

A first dose escalation study for PIPAC-D/C was terminated without dose-limiting toxicity (DLT) and without reaching the maximally tolerated dose (MTD) suggesting an increase by 35% (2.1/10.5) [7]. Most active PIPAC centers adopted this “new” dose without formal validation by a phase II study [8], and the new dose was also accepted for the ISSPP PIPAC course curriculum [9].

Two dose-escalation studies for PIPAC-Ox have been conducted in the same time period in Nantes and Singapore. The French study was terminated at a dose of 90 due to DLTs, while the Singapore study was terminated with the final escalation dose of 120, again without reaching the MTD [10, 11].

The most recent Italian study suggested significantly higher doses without DLTs and without reaching the MTD, namely 135 for PIPAC-Ox and 6/30 for PIPAC-D/C, respectively [12]. However, this study included only a single-shot PIPAC and was terminated prematurely due to administrative limitations. Of note, PIPAC was administered as monotherapy in most of these studies, while in current practice 72% of patients receive PIPAC as add-on treatment embedded in cycles of systemic chemotherapy [8]. Therefore, tolerance and cumulative toxicity might be different for patients under combination therapy and careful consideration is needed before accepting dose increase of the well-established drug regimens. Furthermore, several teams started to add concomitant 5-FU during PIPAC-Ox administration [13]. However, this did not jeopardize tolerance and safety profile in a multi-center retrospective cohort of patients who received ePIPAC in majority combined with systemic chemotherapy [14]. Table 2 provides a comparative overview for the pivotal studies on the different PIPAC-Ox regimens [10], [11], [12].

**Table 2:** Comparison of PIPAC-Ox regimens and their dose-finding studies.

	PIPOX study	NUH study	Turin study
RP2D	90 mg/m^2^	120 mg/m^2^	135 mg/m^2^
Study design	3 + 3	3 + 3	Continual reassessment method
DLT defined	Any grade III or IV toxicity or unexpected post-operative complication.	Any grade 3 toxicity	Not defined
Predefined dose levels	90, 145, 200, 255, 300	45, 60, 90, 120	100, 135, 155
No of included patients	10	17	6
Repeated PIPAC	10	8	No
sCT	Yes	No	No
Grade 3 toxicity	Nausea, neutropenia, anemia, hypersensitivity to Pt, hemorrhage, obstruction	Acute pancreatitis in the first dose level	No
Response at P2RD	PRGS3	PRGS1	NR
Origin of PM	Gastric, CRC + App	Gastric, CRC + App, HPB	Gastric, CRC + App, HPB
Criticism	Hypersensitivity considered as a DLT while it is not dose-dependent	DLT not attained	DLT not clearly defined
	Neutropenia usually excluded from other studies or used as a combined parameter.	Last level not doubled (n=3)	Very limited number of included patients
			Last dose level not doubled (inclusions stopped because of insurance issues)

App, appendiceal cancer; CRC, colorectal cancer; CRM, continual reassessment method; DLT, dose limiting toxicity; HPB, hepato-pancreato-biliary malignancies; NR, not reported; P2RD, phase 2 recommended dose; PRGS, peritoneal regression grading score; sCT, systemic chemotherapy.

Next, new indications for PIPAC treatment have been recently proposed beyond a purely palliative approach. For a neoadjuvant approach with potentially curative approach (secondary cytoreductive surgery ± HIPEC), maximization of treatment effect is the highest priority and higher doses and hence high risk for adverse events (AE) appear acceptable. However, high rate of AEs is inacceptable for PIPAC with prophylactic/adjuvant intention in a setting of unknown benefit for any intraperitoneal chemotherapy [15], [16], [17]. The Odense team proposed therefore to lower ox dose by 50% (46) in their prospective study on prophylactic PIPAC in colorectal cancer patients with high risk for development of PM (NCT03280511) [17].

Finally, other drugs (Nab-paclitaxel for ex.) and drug combinations (Nab-paclitaxel + cis) are under evaluation (NCT03304210, NCT04000906) [18], while others (mitomycin-c (MMC), irinotecan (IRI)) are already used in clinical practice in case of intolerance, allergy etc. Little has been published on these drugs, and different empirical doses are discussed via social media [19].

The aim of this process was to scrutinize currently practiced treatment protocols for PIPAC to reach expert consensus for clinical practice and to identify the most important research questions.

## Methods

The consensus process followed current recommendations and was previously applied to standardize safety and technical aspects of PIPAC treatment [20], [21], [22], [23], [24]:

1. Expert panel: eligible were members of the ISSPP education committee with large personal experience with PIPAC treatment and the principal investigators of prospective studies on PIPAC. The core group (OS, CE, and MH) contacted all eligible persons three times at least and no colleague was deliberately excluded. Requirements for participation were participation in the hybrid consensus meeting and the three-round Delphi process.
2. The core group prepared a comprehensive overview on PIPAC regimens to prepare the consensus meeting including the best available evidence and important confidential information from key opinion leaders.
3. The consensus meeting (Supplementary Material, Appendix 1) was held on July 2nd and 3rd 2021 in Paris and virtual participation (via zoom) was granted according to sanitary requirements due to the COVID-19 pandemic. The program included expert lectures on methodology of dose finding, pharmacology, PIPAC technology and individual presentation of all dose-finding studies by the respective PIs. Controversial aspects were discussed and important research questions were defined. The protocol of the meeting served as basis to prepare the final voting.
4. A modified Delphi approach was used to reach final consensus: The core group prepared a first inquiry based on the evidence. All participants were required to answer the online survey (Survey Monkey, San Mateo, CA, USA, www.momentive.ai) before the consensus meeting. Feedback was provided in the first part of the consensus meeting and a modified version of the survey was repeated *live* with instantaneous feedback (AhaSlides, AhaSlides Pte Ltd, Singapore, ahaslides.com) at the end of the meeting. The core group prepared the final Delphi round based on the discussions and conclusions during the consensus meeting (Supplementary Material, Appendix 2). This final round was sent again to all panelists via Survey Monkey and every participant had four weeks for completion and received four reminders at least.
5. Presentation, discussion and planned validation of the results of the consensus process were planned during the ISSPP bi-annual meeting in Rome, October 7–8th.

The requirements for consensus were an overall response rate higher than 70% and a unidirectional recommendation of more than 70%.

The statistical analysis was mainly descriptive based on the data provided by the web-based platforms (Survey Monkey, AhaSlides). The graphics were prepared by use of Excel software (Microsoft Corp., Redmond, WA, USA).

## Results

Overall, 26 experts were eligible and were invited to participate. Twenty two of them participated in all steps of the consensus process. Nineteen participants were surgical oncologists, two gynecologists and one medical oncologist. 2/22 had additional expertise in pharmacology.

**Figure 1:**
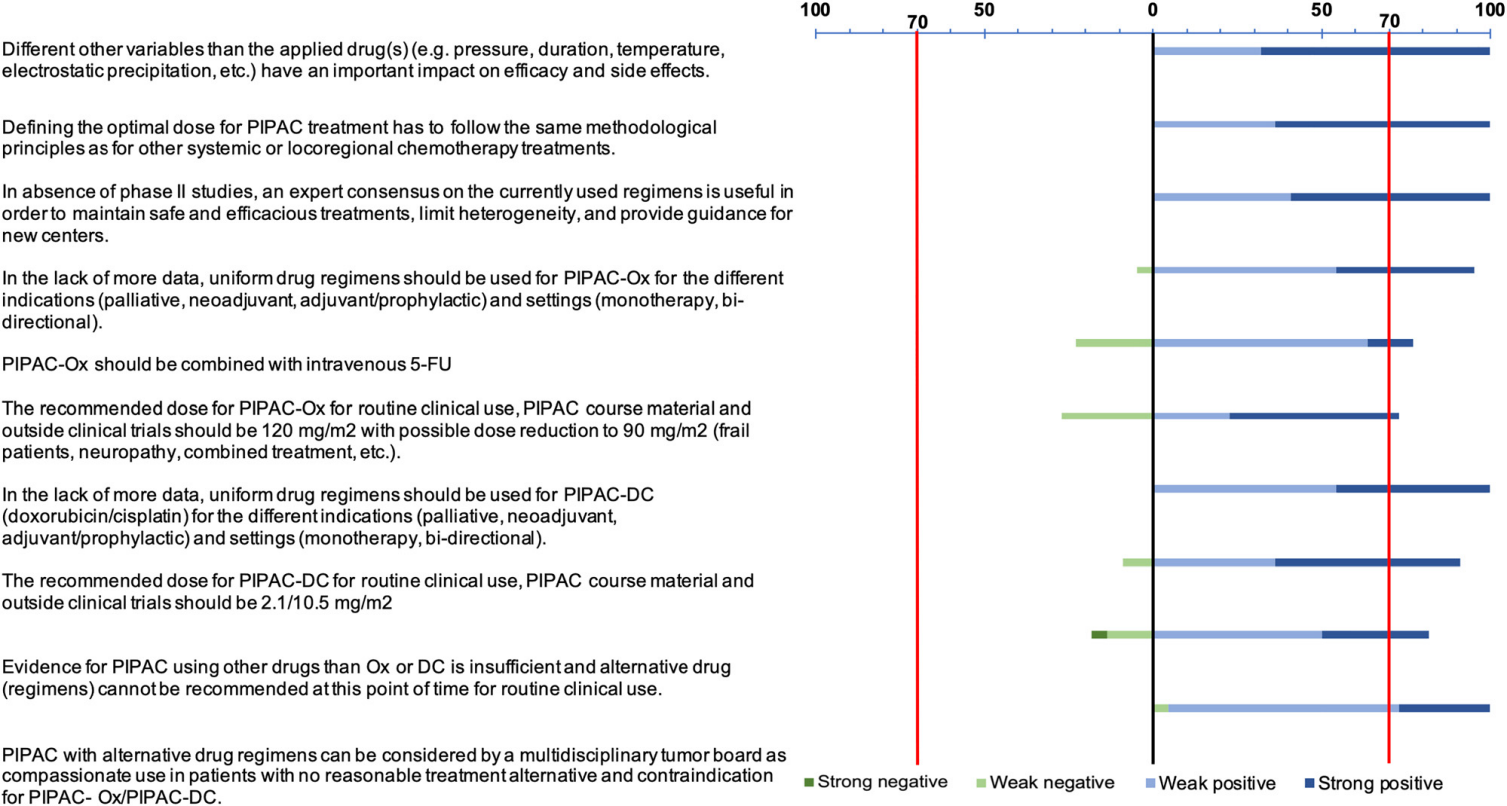
Expert consensus on PIPAC regimens.

Final Delphi three questions (Supplementary Material, Appendix 2) were phrased after expert presentations and in-depth discussion during the two-day hybrid meeting (Supplementary Material, Appendix 1). The panel shared full agreement (100%) for the impact of other variables than drug regimens for PIPAC, endorsement of general principles to define dosing in oncology, but also for the importance of standardization of PIPAC regimens (Figure 1).

There was a strong agreement in favor of uniform PIPAC regimens for the different indications and tumor entities. The combination of doxorubicin (2.1 mg/m^2^) and cisplatin (10.5 mg/m^2^) was endorsed by 20/22 experts (90.9%). 16/22 (72.7%) supported oxaliplatin at 120 with potential reduction to 90 mg/m^2^ (frail patients), and 77.2% suggested PIPAC-Ox in combination with 5-FU (Figure 1). Mitomycin-C and Nab-paclitaxel were favoured as alternative regimens. Suggested dosing for the different drugs are provided as online Supplementary Material, Appendix 3.

**Figure 2:**
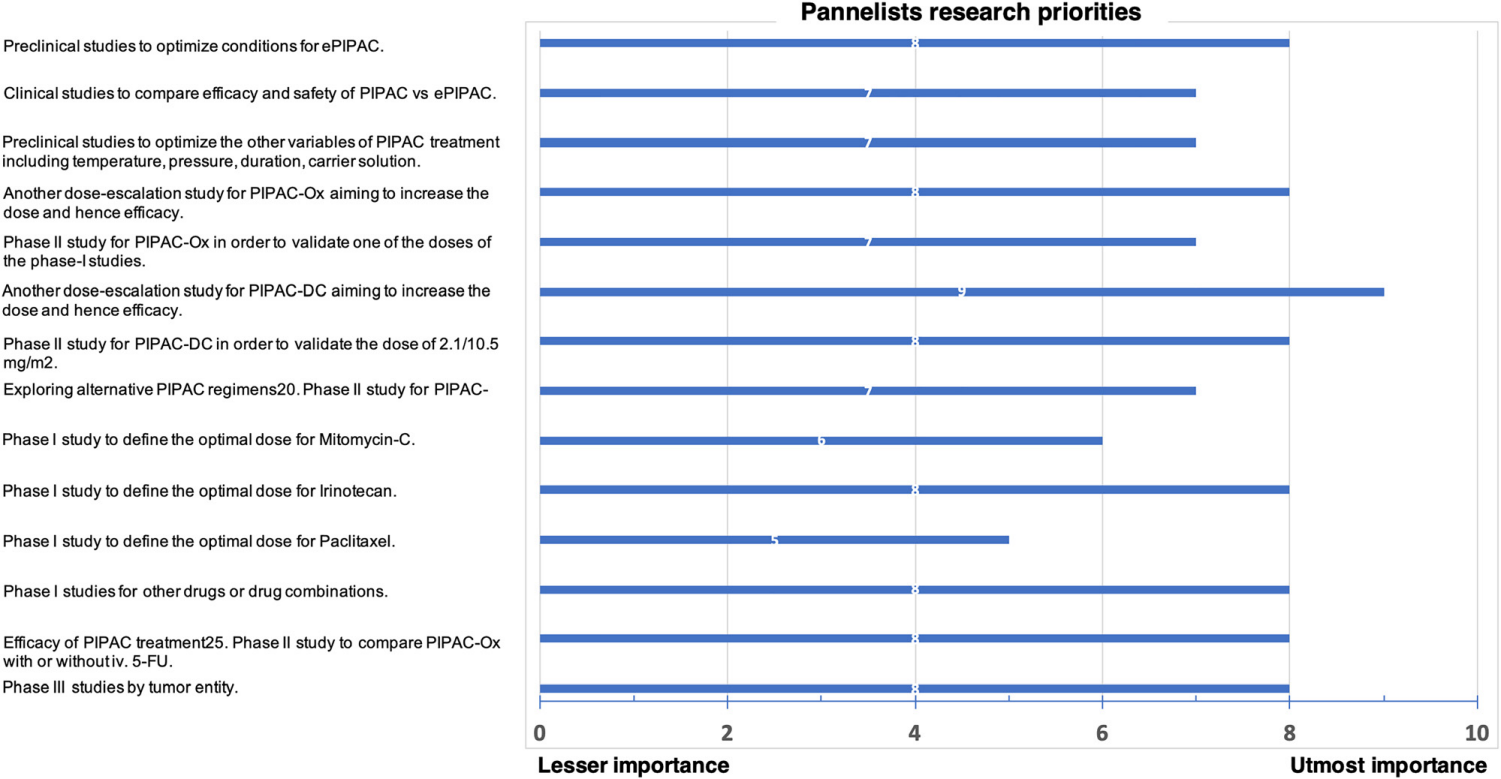
Research priorities to optimize PIPAC treatment. The most important research questions were identified and discussed during the consensus meeting. Panelists rated importance of the topics on a scale from 0 to 10 (highest importance).

The most important research questions concerned PIPAC conditions (n=3), standard (n=4), and alternative regimens (n=5) and efficacy of PIPAC treatment (n=2); eight out of 14 topics were rated to have a priority of ≥8/10 (Figure 2). Of note 10/14 topics were related to drugs and drug regimens.

## Discussion

The expert panel reached consensus on PIPAC regimens for clinical practice while waiting for the results of ongoing studies. Compared to the initial empirical regimens, higher doses were suggested based on the available phase-I studies and concrete alternatives were proposed in case of contraindication. There is high need for further research and the consensus panel identified the most important topics.

The development of PIPAC technology has followed until now the IDEAL stepwise process [25, 26]. Given the number of reported cases and centers [8], it can be considered that an early majority of adopters are now confronted with the diverging results of several phase I trials and they may find difficult to either change the regimen based on conflicting new data or keep the classical regimens they were trained to administer [9]. In order to avoid heterogeneity in the upcoming IDEAL 2b studies and a plethora of PIPAC regimens similar to that of HIPEC regimens [27], it belongs to the experts to compare and synthetize current data in order to select recommended regimens in the waiting of further research.

Phase I trials are a critical step for introducing translational knowledge into clinical practice by establishing the right dose and administration schedule before clinical assessment. They also evaluate the safety of the drug but give very little insight on efficacy [28]. In the traditional Rocket Model of drug development, phase II and III trials are needed in order to grant wide acceptance and integration of new regimens in clinical guidelines [29]. These two steps may require 4–8 years to complete, huge budgets and a waiting time that is often incompatible with the translational science development, resulting in already obsolete data [29, 30]. Rapid development of new regimens, especially in a setting in which there is no present standard treatment, may be based solely on efficacy Phase II trials [29]. That could be the case for PIPAC regimens given that they are often administered in patients with advanced disease that have no standard options of treatment and given that the safety of the procedure is high [4, 7, 10], [11], [12]. Nevertheless, the success of upcoming studies remains dependent on the selection of the best candidate regimen given the various results of the PIPAC phase I trials.

The expert panel analyzed the strengths and the limitations of the different available studies and proposed PIPAC Ox at 120 mg/m^2^ (except in frail patients where 90 mg/m^2^ should be administered) and PIPAC D/C at 2.1/10.5 mg/m^2^. However, new phase I studies and, in particular of PIPAC D/C where the DLT was not met and the study stopped at the highest proposed dose level, were highly recommended as research priorities. The expert group recommended the combination of PIPAC-Ox with 5-FU based on data about the association of the two drugs for HIPEC [13]. Safety and preliminary data for PIPAC-Ox and ePIPAC-Ox exists but the clinical advantage of the association remains to be proved [10, 14, 31].

While homogenous regimens are recommended, there is also a need for more drug options administered as PIPAC, given the large range of primaries responsible for the peritoneal metastases as well as the frequency of allergies to platins and neurotoxicity [8, 32], [33], [34]. Irinotecan and mitomycin C were identified as new priorities in the search of an alternative, particularly in the setting of colorectal cancer.

There is a high interest in ePIPAC that showed impressive distribution and penetration potential in translational studies [35, 36]. While associated morbidity was explored and remains similar to that of traditional PIPAC [14], some of the associated variables (time of exposure, activation of the generator) have still not gained consensus and need further research [23]. This prerogative was confirmed among the research priorities.

The current consensus emerged from a clinical need of PIPAC centers worldwide [8, 23]. In order to deliver recommendations, the expert panel was forced to take into account only current and, thus, limited evidence and to apply a modified Delphi methodology. This approach will probably assist clinicians and help revise the training modules on a medium term but will require itself a rapid updating.

As a consequence, these are neither formal recommendations nor guidelines and several limitations are acknowledged by the authors. The expert panel included mainly surgical oncologists with large PIPAC expertise. Nonetheless, gynecology, medical oncology, and pharmacology were also represented as reflected also by online Supplementary Material, Appendix 1. Due to limited and conflicting evidence, a pragmatic approach and modified Delphi were necessary to reach consensus. A revision of this consensus statement will be necessary as soon as new data is available.

In summary, this consensus aims to avoid diversification of PIPAC regimens to warrant a safe treatment and common standards allowing for prospective multicenter evaluation of its efficacy. Formal evaluation according to the usual standards in oncology need to be pursued and shall help to provide formal recommendations in the future.

## Supplementary Material

Supplementary Material

Supplementary Material

Supplementary Material
