# Supplementary material for: Consensus statement for treatment protocols in pressurized intraperitoneal aerosol chemotherapy (PIPAC)
Source: Pleura Peritoneum. 2022 Mar 1;7(1):1–7. doi: 10.1515/pp-2022-0102 (PMC9069497; doi:10.1515/pp-2022-0102)
Supplement: Supplementary file 1 — Supplementary Material [file j_pp-2022-0102_suppl_001.pdf]

Consensus meeting on PIPAC regimens

Paris 2nd and 3rd of July 2021

Friday, July 2

13:00 Welcome and introduction	M. Hübner
13:15 PIPAC regimens: overview on status quo	O. Sgarbura
13:30 How to define the optimal dose for chemotherapy	W. Pen
14:00 Pharmacology for dummies	K. van der Speeten
14:30 Discussion Marc Pocard	
16:00 PIPAC regimens: how we got started	M.A. Reymond
16:30 Dox/Cis: the optimal dose	C. Tempfer
17:00 Dox/Cis: the optimal dose	M. Robella
17:30 Dox/Cis: status quo	O. Sgarbura
17:45 Discussion	S.P. Somashekhar

Saturday, July 3

9:00 PIPAC: latest news on pressure, temperature, e-PIPAC	M.A. Reymond
9:30 Discussion on PIPAC settings	C. Eveno
10:00 Alternative MMC	M. Alyami
10:15 Alternative Irinotecan, Nab-Palcitaxel	W. Ceelen
10:45 Alternative PIPAC regimens: status quo	O. Sgarbura
11:00 Discussion	W. Ceelen
13:30 PIPAC-Ox finding the optimal dose	
- Pharmacokinetic of e-PIPAC	K.P. Rovers
- Odense	M. Mortensen
- Singapore	J. So
- Turin	M. Robella
- Drug resistance to Oxaliplatin	W. Pen
16:00 PIPAC-Ox: status quo and discussion	O. Sgarbura,
17:00 On-site and online voting on PIPAC drug regimens	D. Cortes Guiral
18:00 Discussion of results and outline of next steps	M. Hübner
