# Supplementary material for: Consensus statement for treatment protocols in pressurized intraperitoneal aerosol chemotherapy (PIPAC)
Source: Pleura Peritoneum. 2022 Mar 1;7(1):1–7. doi: 10.1515/pp-2022-0102 (PMC9069497; doi:10.1515/pp-2022-0102)
Supplement: Supplementary file 2 — Supplementary Material [file j_pp-2022-0102_suppl_002.pdf]

Consensus meeting on PIPAC regimens

Paris 2nd and 3rd of July 2021

General considerations

1. In PIPAC treatment, different other variables than the applied drug(s) have an important impact on efficacy and side effects.

   Strongly agree Agree Disagree Strongly disagree

2. Defining the optimal dose for PIPAC treatment has to follow the same methodological principles as for other systemic or locoregional chemotherapy treatments.

   Strongly agree Agree Disagree Strongly disagree

3. In absence of phase II studies, an expert consensus on the currently used regimens is useful in order to maintain safe and efficacious treatments, limit heterogeneity and to provide guidance for new centers.

   Strongly agree Agree Disagree Strongly disagree

Standard PIPAC dose regimens

4. In the lack of more data, uniform drug regimens should be used for PIPAC for the different indications (palliative, neoadjuvant, adjuvant/prophylactic) and settings (monotherapy, bi-directional).

   Strongly agree Agree Disagree Strongly disagree

5. The recommended dose for PIPAC-Ox for routine clinical use / PIPAC course material and outside clinical trials is …

   a. 46 90 92 120 135

      other (free text) do not use PIPAC-Ox

   b. PIPAC-Ox should be combined with 5-FU

   Strongly agree Agree Disagree Strongly disagree

6. The recommended dose for PIPAC-Ox for routine clinical use / PIPAC course material and outside clinical trials should be 120 with possible dose reduction to 90 (frail patients, neuropathy, combined treatment etc.)

   Strongly agree Agree Disagree Strongly disagree

7. The recommended dose for PIPAC-DC for routine clinical use / PIPAC course material and outside clinical trials should be 2.1/10.5

   Strongly agree Agree Disagree Strongly disagree

Alternative drug regimens

8. Evidence for PIPAC using other drugs than Ox of DC is insufficient and alternative drug (regimens) can**not** be recommended at this point of time for routine clinical use.

Strongly agree Agree Disagree Strongly disagree

9. PIPAC with alternative drug regimens **can** be considered by a multidisciplinary tumor board as compassionate use in patients with no reasonable treatment alternative and contraindication for PIPAC-Ox/PIPAC-DC.

   Strongly agree Agree Disagree Strongly disagree (no PIPAC possible in this patient)

10. In the absence of dose-escalating studies and as compassionate use as outlined above, the following drug regimen appears to be a reasonable choice according to the limited evidence and expert opinion:

    a. Mitomycin-C

    1.5 mg/m^2 8 mg/m^2 14 mg total dose (other) should **not** be used

    b. Irinotecan (mg/m^2)

    20 (other) should **not** be used

    c. Paclitaxel (mg/m^2)

    30 (other) should **not** be used

    d. Nab-Paclitaxel (mg/m^2)

    112.5 (other) should **not** be used

Research priorities:

Please rate the importance on a VAS from 0 (not important) to 10 (maximal importance)

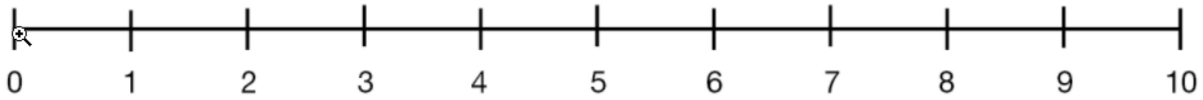

PIPAC conditions

11. Preclinical studies to optimize conditions for ePIPAC
12. Clinical studies to compare efficacy and safety of PIPAC *vs* ePIPAC
13. Preclinical studies to optimize the other variables of PIPAC treatment including temperature, pressure, duration, carrier solution …

Optimizing standard PIPAC regimens

14. Another dose escalation study for PIPAC-Ox aiming to increase dose and hence efficacy.
15. Phase II study for PIPAC-Ox in order to validate one of the doses of the phase-I studies (optional free text: dose)
16. Another dose escalation study for PIPAC-DC aiming to increase dose and hence efficacy.
17. Phase II study for PIPAC-DC in order to validate the dose of 2.1/10.5

Exploring alternative PIPAC regimens

18. Phase II study for PIPAC-Nab paclitaxel in order to validate the dose of 112.5
19. Phase I study to define the optimal dose for MMC
20. Phase I study to define the optimal dose for IRI
21. Phase I study to define the optimal dose for Paclitaxel
22. Phase I studies for other drugs or drug combinations (optional free text: which one(s))

Efficacy of PIPAC treatment

23. Phase II study to compare PIPAC-Ox with or without 5-FU

24. Phase III studies by tumor entity
