# Supplementary material for: Consensus statement for treatment protocols in pressurized intraperitoneal aerosol chemotherapy (PIPAC)
Source: Pleura Peritoneum. 2022 Mar 1;7(1):1–7. doi: 10.1515/pp-2022-0102 (PMC9069497; doi:10.1515/pp-2022-0102)

Different **other variables than the applied drug(s)** have an important impact on **efficacy** and **side effects**.

Defining the **optimal dose** for PIPAC treatment has to follow **pharmaco-clinic methodological principles**.

In absence of phase II studies, an **expert consensus** on the currently used regimens **is useful** in order to maintain safe and efficacious treatments, limit heterogeneity, and provide guidance for new centers.

In the lack of more data, **uniform drug regimens** should be used for **PIPAC-Ox** for the different indications (palliative, neoadjuvant, adjuvant/prophylactic) and settings (monotherapy, bi-directional).

PIPAC-Ox should be **combined with intravenous 5-FU**

The recommended dose for **PIPAC-Ox should be 120 mg/m2** with possible dose reduction to 90 mg/m2 (frail patients)

In the lack of more data, **uniform drug regimens** should be used for **PIPAC-D/C** (doxorubicin/cisplatin) for the different indications (palliative, neoadjuvant, adjuvant/prophylactic) and settings (monotherapy, bi-directional).

The recommended dose for **PIPAC-D/C should be 2.1/10.5 mg/m2**

Evidence for PIPAC **using other drugs than Ox or D/C** is insufficient and alternative drug (regimens) **cannot be recommended** at this point of time for routine clinical use.

PIPAC with **alternative drug regimens** can be considered by a **multidisciplinary tumor board** as **compassionate use** in patients with no reasonable treatment alternative and contraindication for PIPAC- Ox/PIPAC-DC.

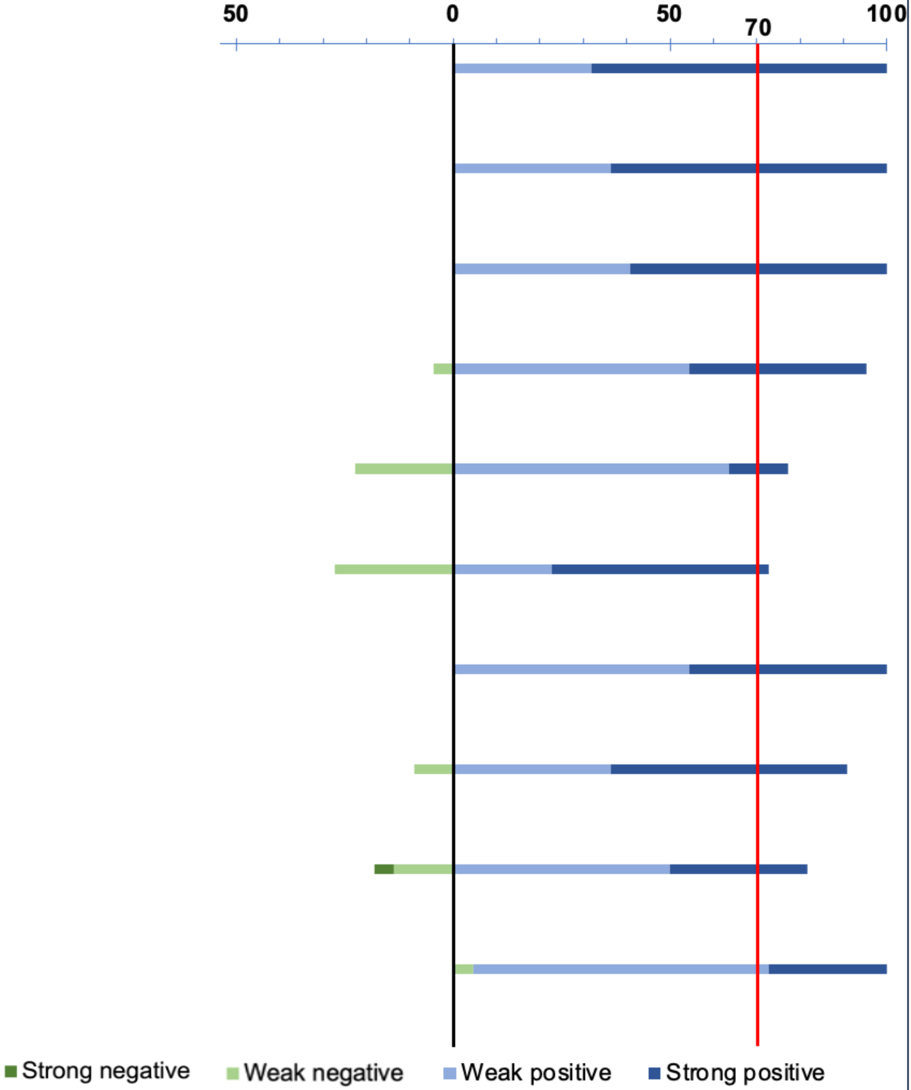

Supplement: Supplementary file 3 — Supplementary Material [file j_pp-2022-0102_suppl_003.pdf]
